# Solitary fibrous tumor of the tongue

**DOI:** 10.4322/acr.2021.405

**Published:** 2022-11-17

**Authors:** Juliana Mota Siqueira, Daniele Heguedusch, Emília Maria Gomes Aguiar, Anaeliza Figueiredo dos Santos, Fabio Abreu Alves, Fabio Daumas Nunes

**Affiliations:** 1 Universidade de São Paulo (USP), Dental School, Department of Oral and Maxillofacial Pathology, São Paulo, SP, Brasil; 2 Universidade de São Paulo (USP), Dental School, Stomatology Department, São Paulo, SP, Brasil; 3 A.C. Camargo Cancer Center, Stomatology Department, São Paulo, SP, Brasil

**Keywords:** Solitary Fibrous Tumors, Oral diagnosis, Oral Pathology

## Abstract

Solitary fibrous tumor (SFT) is a benign mesenchymal neoplasm originally described in pleura with a rare presentation in the oral cavity. Herein, we report a case of a 28-year-old male patient who presented an asymptomatic slow-growing mass in the anterior part of the tongue. Intraoral examination revealed a well-circumscribed mass covered by normal mucosa with a fibrous consistency. Due to non-specific clinical findings, the initial diagnostic hypotheses include benign submucosal neoplasms such as leiomyoma, neurofibroma, SFT, and others. An excisional biopsy was performed. Microscopically, the tumor was surrounded by a thick fibrous capsule; hypo and hypercellular areas were arranged in a storiform pattern with a stroma formed by collagen and abundant vascularization. Tumor cells showed immunopositivity for CD34 and STAT-6 and no expression of CD99, AML, S-100, and Ki-67. According to these findings, the diagnosis of SFT was established. After 24 months, the patient is asymptomatic and has no evidence of recurrence. Although oral involvement is rare, SFT should be included in the differential diagnosis of oral submucosal lesions.

## INTRODUCTION

Representative of rare mesenchymal neoplasms, solitary fibrous tumor (SFT) is a tumor of fibroblastic/myofibroblastic origin that occurs in different parts of the body. Its first morphological description was performed in pleural tumors in 1931 by Klemperer and Rabin, but it was not until 1990 that this lesion was reported in extra-thoracic sites.^1^ The head and neck region comprise nearly 5% of all SFT occurrences, mostly reported in the oral cavity. In this setting, the buccal mucosa, tongue, palate, and floor of the mouth have been the most involved regions, respectively.^2^,^3^ SFT affects mainly adults during the fifth decade of life and has no sex predilection. Further, factors that are associated with its development are poorly understood.^4^

Clinically, this lesion appears as a slow-growing, indolent nodule covered by normal mucosa and can be easily confused with reactive lesions or other neoplasms that affect the oral cavity.^5^ Microscopic findings show a well-delimited lesion displaying alternating areas of hypercellularity and hypocellularity in a collagenous to the myxoid stroma. Individually, cells may exhibit spindle-to ovoid-shaped with indistinct cytoplasmic borders distributed in small fascicles or arranged in a disorganized pattern known as a "patternless pattern." The staghorn-shaped blood vessel presence and perivascular hyalinization are also important findings for SFT diagnosis.^6^

Because they present, histopathological features that overlap with other lesions, diagnosis of SFT must be performed in combination with immunomarkers expression.^7^ Recent studies have demonstrated the presence of a chromosomal fusion (NAB2-STAT6) in many SFT cases.^8^ Thus, coherent nuclear immunoexpression of STAT6 protein has been used with high sensitivity and specificity, together with CD44, to diagnose SFT. Other markers such as BCL-2, and CD99 are also part of this immunohistochemical panel; however, due to their low specificity, they are not considered predictive in the diagnosis of SFT.^9^

The latest World Health Organization (WHO) Soft Tissue and Bone Tumors classification has considered the biological behavior of SFT as intermediate due to its ability to recur locally and promote local or distant metastases in a minority of cases.^10^ This aggressive profile has been associated with cellular/nuclear pleomorphism, hypercellularity, mitosis, necrosis, and infiltrative growth. Thus, the identification of these morphological parameters may suggest SFT malignancy.^11^ Taking into account the rarity of the lesion and its uncertain biological behavior, reports in the literature and patient follow-up are extremely necessary. This study aimed to present the diagnosis process and management of a SFT case in the tongue region of a young patient.

## CASE REPORT

**Figure 1:**
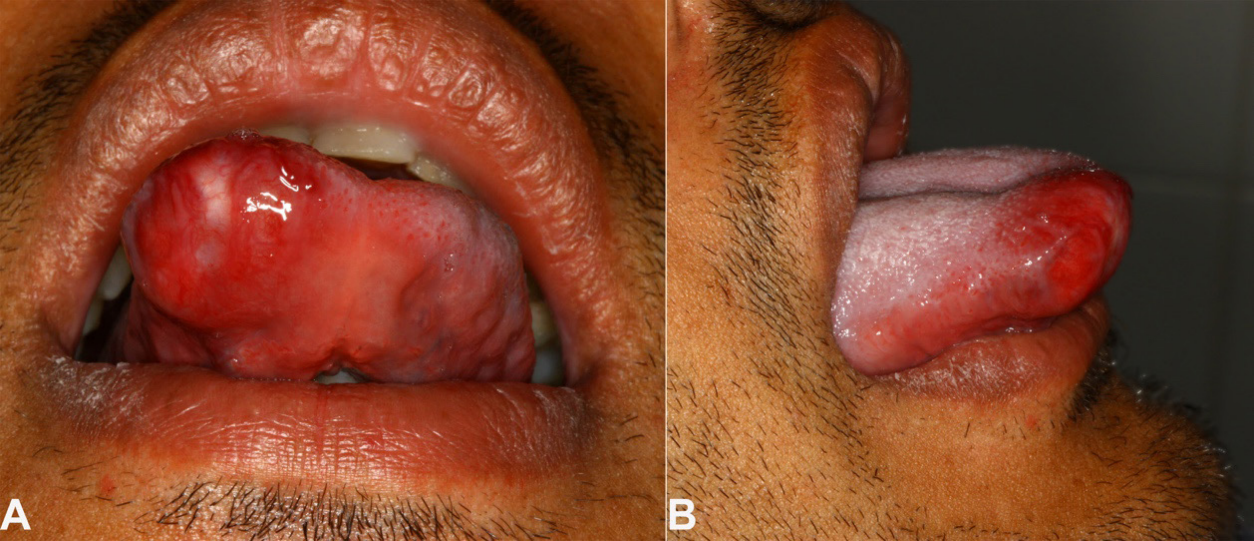
Intraoral examination shows: **A** - a well-circumscribed mass covered by an erythematous mucosa in the anterior portion of the tongue (Frontal view); **B** - right lateral view.

A 28-year-old patient presented to the Stomatology Department complaining of an asymptomatic slow-growing mass in the tongue over the past 3 months. An intraoral examination revealed a well-delimited nodule with fibrous consistency in the anterior portion of the tongue, measuring 2.0 cm in the maximum dimension. The lesion was covered by erythematous surface mucosa (Figures 1A, and 1B).

**Figure 2:**
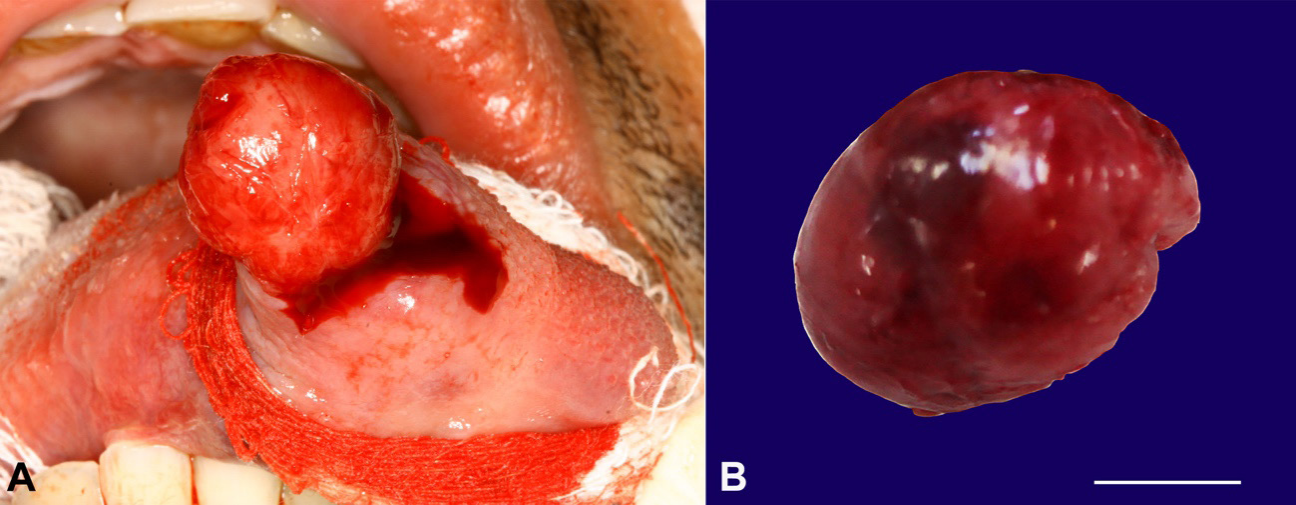
**A** - Trans-operative aspect of encapsulated lesion on the submucosa; **B** - size specimen (scale bar = 1 cm).

According to the clinical findings, the diagnosis was suggestive of benign mesenchymal tumors such as leiomyoma, neurofibroma, and SFT. Surgical excision revealed an encapsulated lesion on the submucosa, which supported our previous hypothesis of a benign tumor (Figures 2A and 2B).

**Figure 3:**
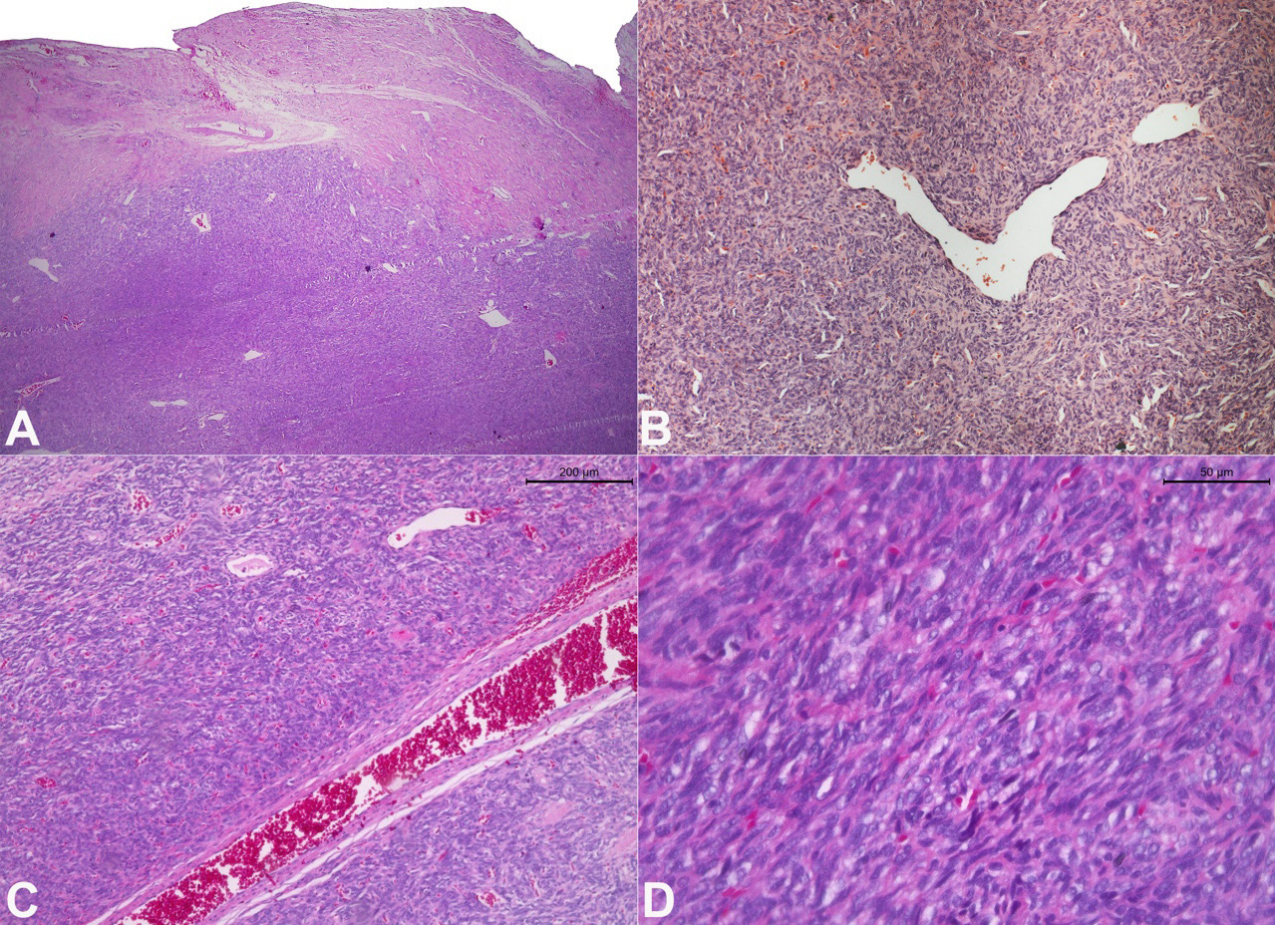
Photomicrographs of the biopsy specimen show: **A** - a dense proliferation of neoplastic cells partially surrounded by a thick fibrous capsule (H&E, 2.5X); **B** - Staghorn-like vessels (H&E, 10X); **C** - branching with evident perivascular hyalinization (H&E, 10X); **D** - Sheet of the spindle to ovoid cells with indistinct cytoplasmic borders (H&E, 40X).

The histological examination evidenced a dense proliferation of neoplastic mesenchymal cells partially surrounded by a thick fibrous capsule (Figure 3A). Hypo and hypercellular areas were arranged in a storiform pattern with a stroma formed by thin collagen fibrils and abundant vascularization. Staghorn-like vessels (Figure 3B) and branching with evident perivascular hyalinization can be identified (Figure 3C). At high power, tumor cells showed a spindle to ovoid shape with indistinct cytoplasmic borders and a small number of mitotic features (Figure 3D).

**Figure 4:**
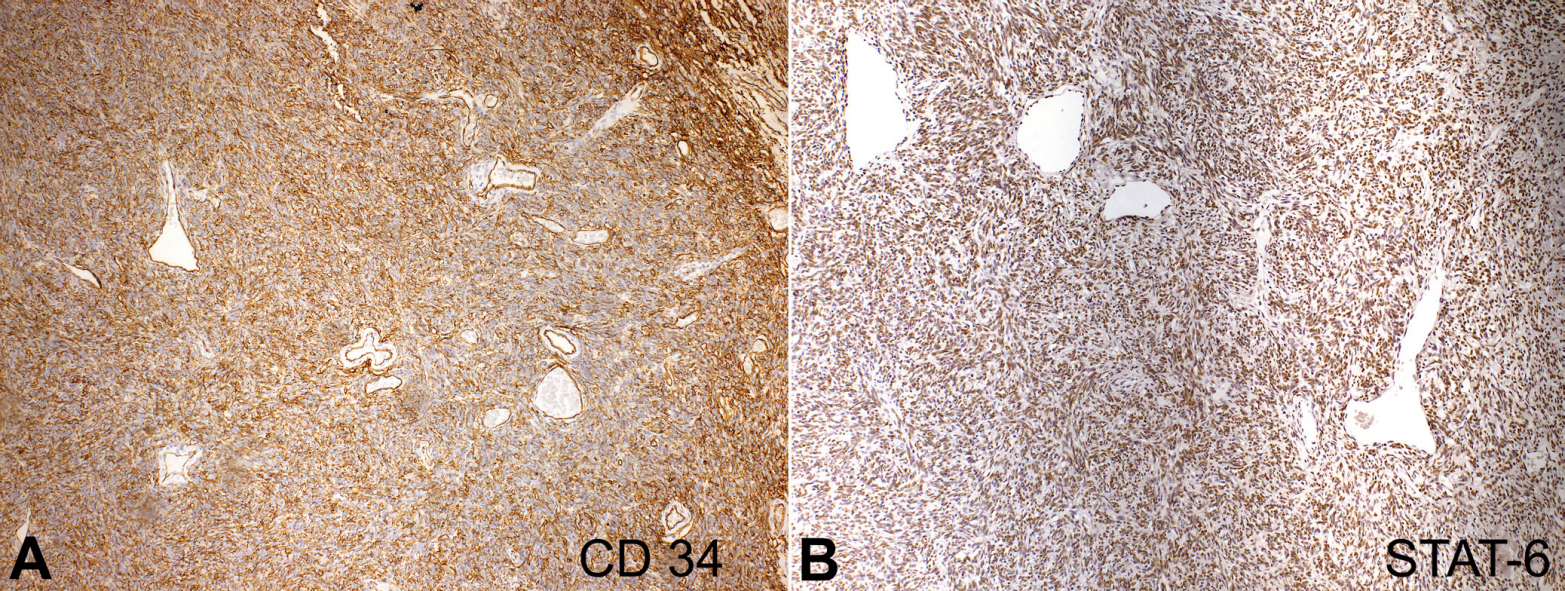
Immunohistochemical panel showing the neoplastic cells with: **A** - diffuse expression of CD34 (10X); **B** - STAT-6 (10X).

The tumor cells showed immunopositivity for CD34 (Figure 4A), STAT-6 (Figure 4B), and no expression of CD99, AML, S-100, and Ki-67. According to these findings, the diagnosis of the solitary fibrous tumor was established. After 24 months of follow-up, no recurrence has been detected.

## DISCUSSION

Solitary fibrous tumor (SFT), first reported in the pleura,^1^ has been diagnosed in different anatomical sites, including the oral cavity.^2^,^3^ Although extremely rare at this site, SFT can occur in several regions of the oral cavity, including buccal mucosa, vestibule, lip, tongue, gingiva, and alveolar mucosa.^2^,^3^,^12^ Different from our case, SFT is more common in the fifth decade of life, and as far as we know, there is no sex predilection. In addition, the factors that led to its emergence are not fully understood.^4^

Clinically SFT is characterized by a submucosal nodule, well-circumscribed or encapsulated, painless, slow-growing, and variable in size.^5^ Our report case shares similar features. Trans-operative procedure confirmed circumscribed lesion with fibrous capsule. Furthermore, the final diagnosis was performed based on clinical, histopathological and immunohistochemical characteristics.

It is recognized that most SFT cases present immunopositivity for CD34. When it is negative, the diagnosis becomes more challenging. Therefore, the use of other markers such as CD99 and STAT6 is highly recommended.^5^,^13^ In our results, immunohistochemical analysis showed positive labeling for CD34. This glycoprotein is a cell adhesion factor that can be found in different neoplasms; in SFT its expression is observed in 81-95% of cases.^14^ As mentioned above, CD99 has been used as an alternative marker for SFT diagnosis, but its sensitivity rate is lower (75%).^9^ In our case, CD99 was negative, as pointed out in other studies.^13^ Thus, although CD99 has been described as an important alternative marker for SFT, it cannot be considered a predictor for diagnosis. 3

Recent evidence shows that NAB2-STAT6 gene fusions were found by the nuclear expression of the STAT6 protein in SFT cases, which has now become the gold standard marker for diagnosis due to its high sensitivity and specificity. It is also suggested that SFT development may be related to this fusion of genes induced by 12q chromosome rearrangement.^15^ A systematic review shows that both expressions of CD34 and STAT6 were found to be positive in totally of SFT cases, which agrees with our results showing nuclear expression of STAT6 and positivity for CD34.^3^

In addition, SFT frequently shows negativity for other well-known markers involved in the differential diagnosis of soft-tissue lesions, such as cytokeratin, α-SMA, and S-100 protein.^5^,^16^ In our case report, immunohistochemical analyses were also negative for protein S-100 and α-SMA. Finally, since SFT displays an intermediate biological behavior, local recurrence and distant metastases may occur in rare cases. Cellular/nuclear pleomorphism, hypercellularity, mitosis, necrosis, and infiltrative growth are normally related to this aggressive profile.^10^,^17^ In this context, it is helpful to verify the proliferation marker, Ki-67. It is known that approximately 10% of SFT cases show positivity for Ki-67.^16^ Here, we showed a negative result for the Ki-67 marker, due to the absence of mitosis and nuclear atypia, which is similar to other reports in the literature.^18^

In conclusion, although SFT is rare, it should be included in the diagnostic hypothesis of submucosal soft-tissue oral lesions. A complete study of clinical, histopathological and immunohistochemical characteristics is essential for diagnosing and appropriately managing these cases.
